# Left subclavian artery bridging stent fracture after in-situ laser fenestration during emergent thoracic endovascular aortic repair

**DOI:** 10.1016/j.jvscit.2024.101550

**Published:** 2024-06-05

**Authors:** Austėja Račytė, Luis H. Arzola, Anders Wanhainen, Giuseppe Asciutto, Marek Kuzniar, Kevin Mani

**Affiliations:** a Department of Surgical Sciences, Section of Vascular Surgery, Uppsala University, Uppsala, Sweden; b Faculty of Medicine, Vilnius University, Vilnius, Lithuania; c Department of Surgical and Perioperative Sciences, Surgery, Umeå University, Umeå, Sweden

**Keywords:** In-situ laser fenestration, Stent fracture, Thoracic aortic aneurysm

## Abstract

In-situ laser fenestration (ISLF) has been described as a viable option for urgent thoracic aortic aneurysm cases involving supra-aortic vessels. There are, however, limited data on its durability. Here, we present a case of a 70-year-old man with a symptomatic 13-cm thoracic aortic aneurysm extending proximally to the origin of the left subclavian artery (LSA). Emergent thoracic endovascular aortic repair with chimney stenting of the left common carotid artery and ISLF for the LSA was successfully performed. During the follow-up, a compression of the bridging stent to the LSA progressed to a stent fracture needing realignment. Despite ISLF’s reported technical success, this case highlights the risk of bridging stent complications, emphasizing the need for a close follow-up.

Despite the ongoing progress in endovascular surgery, complex thoracic aortic aneurysms (TAAs) involving the supra-aortic vessels still pose a technical challenge,^1^^,^^2^ particularly in acute settings. Traditionally, such situations require complex hybrid solution with a thoracic endovascular aortic repair (TEVAR) and selective extraanatomical bypass reconstruction.^1^^,^^3^

The in-situ laser fenestration (ISLF) technique^4^^,^^5^ is a valuable option in the acute setting in selected patients, such as in the case of patients with hostile neck. Despite its appeal, data on its long-term efficacy are lacking. Herein, we present a case of bridging stent fracture following ISLF procedure during TEVAR successfully treated endovascularly. The patient has given an informed consent for the publication of this manuscript.

## Case report

**Fig 1:**
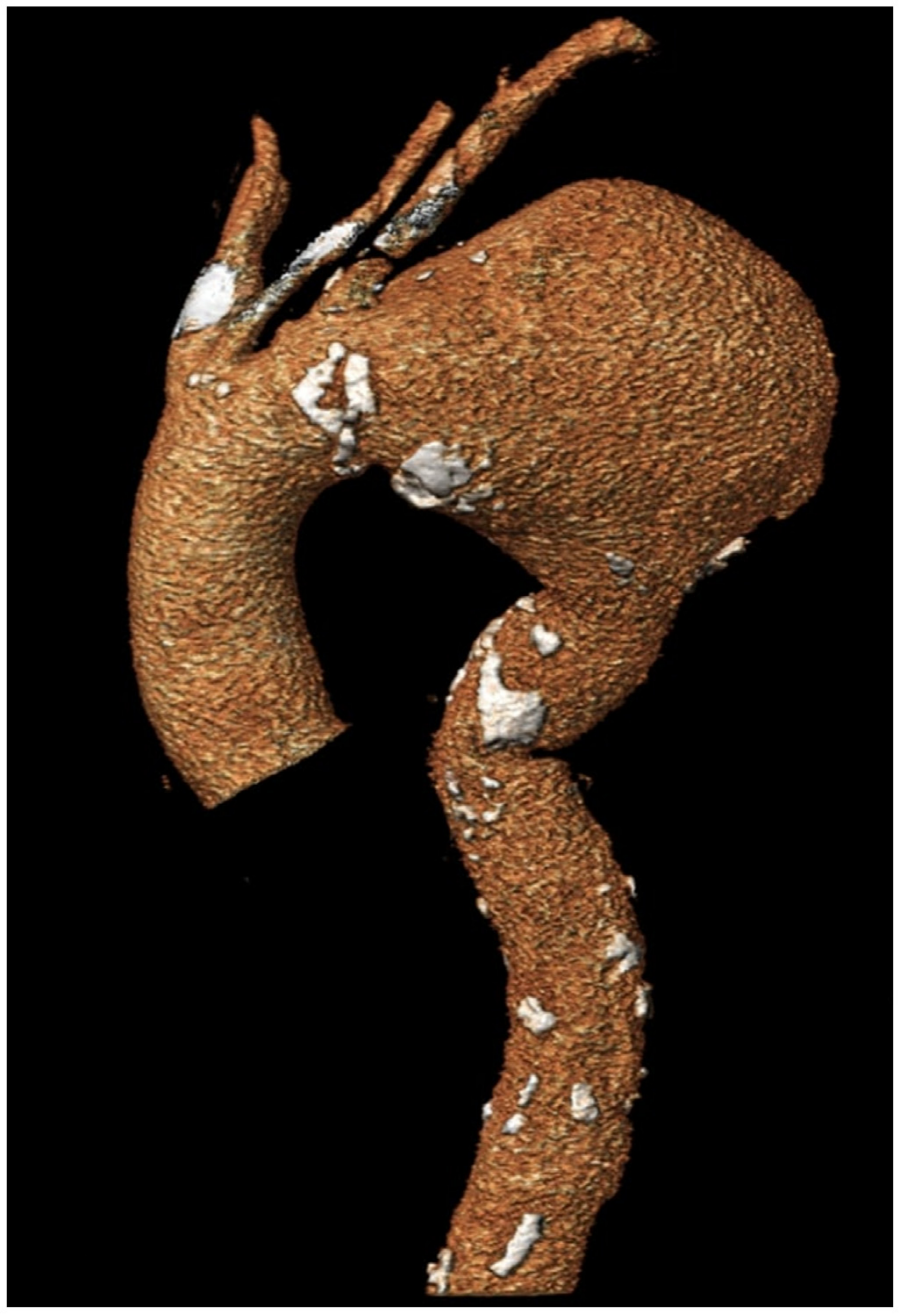
Preoperative three-dimensional reconstructions of 13-cm thoracic aortic aneurysm (*TAA*) that was treated with implantation of a thoracic stent graft with proximal landing with partial coverage of the left common carotid artery (*LCCA*) and the left subclavian artery (*LSA*). The LCCS was revascularized with a chimney stent graft, and the LSA with in situ laser fenestration (*ISLF*) and stent graft implantation in this fenestration.

A 70-year-old active smoker without any prior surgical history nor a known family history for aortic diseases presented to the emergency department with a 2-week history of intermittent chest pain, hoarseness, and dry cough. Computed tomography (CT) angiography revealed a 13-cm TAA with left-side pleural effusion (Fig 1). The patient had a bovine configuration with a type II arch. The landing zone length between the left common carotid artery (LCCA) and left subclavian artery (LSA) was 13 mm measured on centerline and 18 mm on the outer, as well as 13 mm on the inner curvature. The left vertebral artery was chronically occluded. Given the risk of impending rupture and the anatomical configuration of the patient’s vessels, the patient was scheduled for an urgent TEVAR with ISLF for the LSA and a chimney for the LCCA. In this particular case, the chimney technique for LCCA was used due to the patient’s bovine arch with a large common origin of the brachiocephalic trunk and LCCA and the early takeoff of the LCCA from this common origin. The chimney technique allowed maintaining antegrade flow to both arteries without resorting to open surgery on the neck or chest.

The ISLF technique has been previously described by several authors.^5^, ^6^, ^7^ Briefly, with the patient under general anesthesia, open exposure of the left brachial and left carotid arteries was performed, while the common femoral artery was accessed percutaneously bilaterally. Additionally, access to the left femoral vein for caval occlusion was obtained.

Cook Zenith Alpha tapered (42 mm-38 mm-173 mm) endograft and COOK Zenith Alpha distal component (38 mm-38 mm-197 mm) (Cook Medical LLC) were introduced through the right femoral access and deployed under caval occlusion. Caval occlusion was performed using Coda balloon catheter (Cook Medical LLC). It was inserted into the inferior caval vein through the femoral vein and inflated before deploying the stent graft. Caval occlusion was used to decrease aortic pressure and flow, which minimized the potential downstream shift of the stent graft.

**Fig 2:**
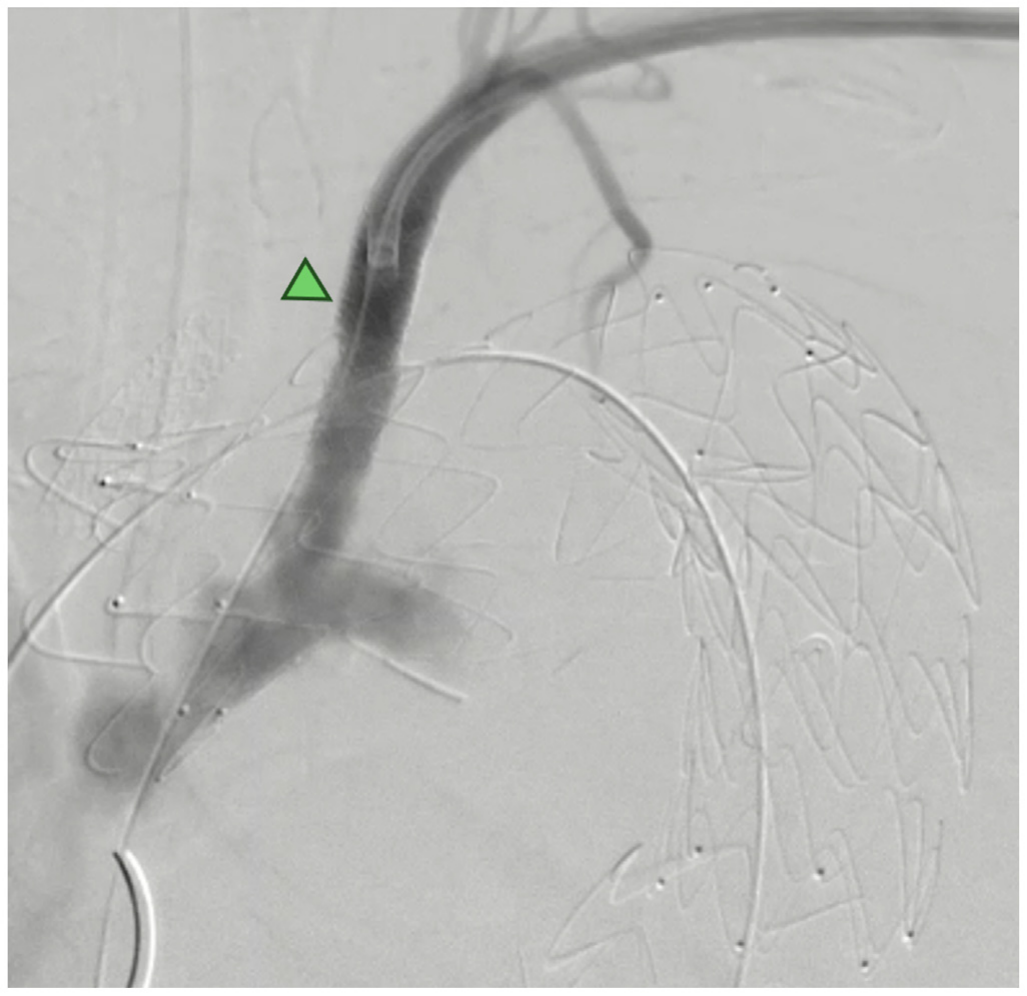
Completion angiography demonstrating a fully patent left subclavian artery (*LSA*) bridge stent after in situ laser fenestration (*ISLF*) technique (*green arrowhead*).

An 8-mm Advanta V12 (Atrium) was deployed in the LCCA using the chimney graft technique. Subsequently, a steerable sheet was used to access the LSA, and an ISLF was performed with a 1.7-mm Turbo-Elite Laser Catheter (Philips), with an 0.018 wire passing into the ascending aorta through the ISLF fenestration. The fenestration was ballooned up to 5 mm afterwards. After the creation of the ISLF, a 9-mm Gore Viabahn VBX Balloon Expandable Endoprosthesis (W. L. Gore & Associates) was deployed. Completion angiography revealed normal flow to the LSA and LCCA, with complete endovascular exclusion of the TAA without any visible signs of stent compression or endoleaks (Fig 2). The patient was discharged after 7 days with single antiplatelet therapy (ASA) and without any complications.

**Fig 3:**
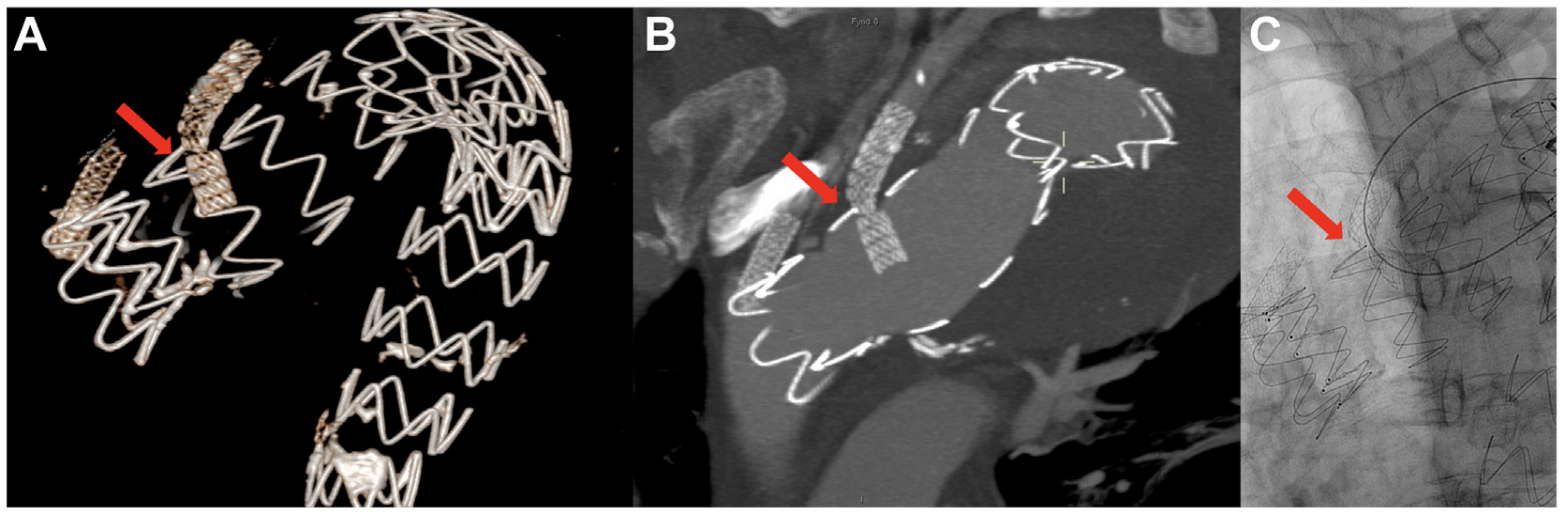
**A,** Three-dimensional reconstruction of the fractured left subclavian artery (*LSA*) bridging stent (*red arrow*). **B,** Two-month computed tomography (*CT*) scan showing significant stenosis of the LSA bridging stent graft (*red arrow*). **C,** Intraoperative image of the fractured bridging stent graft (*red arrow*).

**Fig 4:**
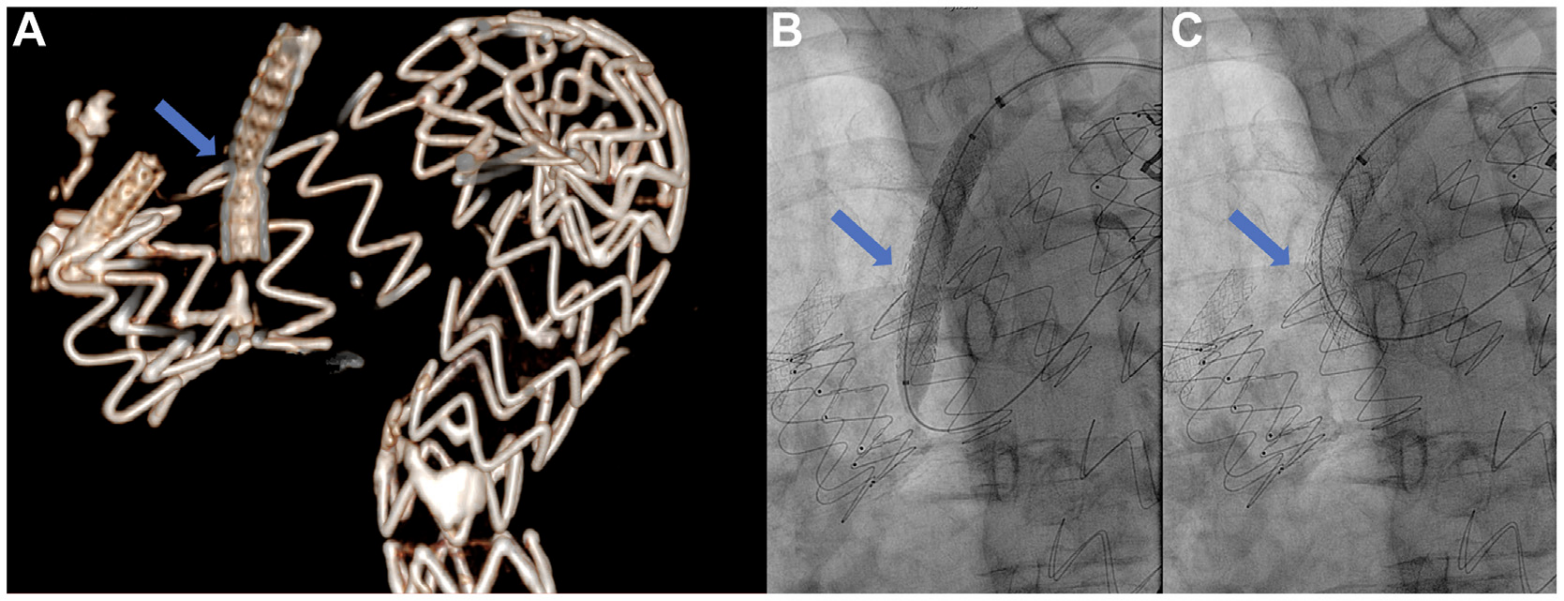
**A-C,** Left subclavian artery (*LSA*) bridging stent fracture was re-lined with a 9-mm Advanta V12 (*blue arrows*).

The first follow-up CT angiography 2 months postoperatively showed a significant stenosis of the LSA bridging stent graft (Fig 3, *A* and *B*). The patient was scheduled for a reintervention. Imaging at time of reintervention showed a fully fractured LSA stent (Fig 3, *C*). Successful cannulation of the LSA was performed through left brachial access, and the stent graft was realigned with a 9-mm Advanta V12 (Fig 4). Completion angiography showed good flow to the LSA and no visible endoleaks. The patient had uneventful recoveries after both procedures, and no spinal cord protection measures were necessary. The patient underwent a follow-up CT scan 2 months after the reintervention and had a clinic visit a month later without any signs of restenosis.

## Discussion

Stent graft fracture has been previously described as a possible source of endoleak and target vessel instability after complex aortic endovascular reconstructions with custom-made devices.^8^ According to bench-testing of aortic endografts, performed by Grima et al, Dacron endografts have the most favorable results with ISLF.^6^

In-situ fenestration is considered an alternative endovascular option when aortic pathologies involve the supra-aortic vessels.^9^^,^^10^ Complication rates for ISLF are considered to be relatively low, and they mostly include failure of fenestration, endoleaks (mostly type III), and dissection of target vessels.^10^

In this paper, we describe the first case of bridging stent graft fracture after ISLF during TEVAR. According to existing literature, balloon expandable stents are more rigid and straight, which makes them more susceptible to collapse and fractures when the external pressure is high.^11^^,^^12^ The non-reinforced character of the ISLF makes the bridging stent grafts theoretically more prone to instability. One of the possible mechanics advocated is that of a direct compression due to the metal struts of the TEVAR stent graft. This could lead to a considerable mechanical stress in the region of the ISLF. A systematic analysis conducted by Prendes et al suggests the use of multifilament polyethylene terephthalate, followed by dilation with noncompliant balloons, as the most durable in vitro technique for ISLF.^5^

In this particular case, the Gore Viabahn VBX Balloon Expandable Endoprosthesis configuration, consisting of individual stent rings with PTFE graft in-between, could have contributed to its instability through the ISLF.

Considering the unexpected complication that occurred after the ISLF during this emergent TEVAR, an individualized follow-up plan was utilized for this patient. The first CT scan was performed 2 months after the reintervention, with a yearly CT scan being planned. Any target vessel instability seen during the follow-up scan would be addressed accordingly. The open conversion (ie, a carotid-subclavian bypass or a total debranching based on the patency of the chimney to the left common carotid artery) could be considered as an option.

## Conclusion

ISLF is a promising technique allowing rapid customization of TEVAR stent graft for acute treatment of aortic arch pathology that requires supra-aortic vessel revascularization. The current report, however, underlines the risk for bridging stent graft instability with this new technique. Close postoperative follow-up is advocated after ISLF treatment of aortic arch pathology, with dedicated evaluation of bridging stent graft integrity with CT imaging.

## Disclosures

K.M reports institutional grant from and consulting for Cook Medical Inc. A.W. reports institutional grant from 10.13039/100010479Cook Medical Inc.
